# Performance of deamidated gliadin peptide antibodies as first screening for celiac disease in the general pediatric population

**DOI:** 10.3389/fped.2023.1279825

**Published:** 2023-11-21

**Authors:** Abdulrahman Al-Hussaini, Abdullah Al-Jurayyan, Sahar Alharbi, Muhammed Salman Bashir, Riccardo Troncone

**Affiliations:** ^1^ Division of Pediatric Gastroenterology, Children’s Specialized Hospital, Riyadh, Saudi Arabia; ^2^ College of Medicine, Alfaisal University, Riyadh, Saudi Arabia; ^3^ Prince Abdullah Bin Khaled Celiac Disease Research Chair, Department of Pediatrics, Faculty of Medicine, King Saud University, Riyadh, Saudi Arabia; ^4^ The Department of Pathology and Clinical Laboratory Medicine, Immunology, Serology & HLA Laboratory Section, King Fahad Medical City, Riyadh, Saudi Arabia; ^5^ Department of Biostatistics, Research Services Administration, Research Center at King Fahad Medical City, Riyadh, Saudi Arabia; ^6^ Department of Medical Translational Sciences & European Laboratory for the Investigation of Food-Induced Diseases, University Federico II, Naples, Italy

**Keywords:** celiac disease, deamidated gliadin peptides, tissue transglutaminase, mass screening, Saudi Arabia

## Abstract

**Background:**

Celiac serology has evolved, with the identification of newer antibodies against deamidated gliadin peptides (DGP) [e.g., anti-DGP, immunoglobulin A (IgA), and immunoglobulin G (IgG) types] with sensitivity and specificity in detecting celiac disease (CeD) that are equivalent to anti-tissue transglutaminase [anti-tissue transglutaminase (TTG) IgA]-based tests, particularly in populations with high pretest probability of CeD (prevalence of CeD > 50% of the population under study). This opens the possibility that anti-DGP assays can be used to identify CeD in the general population where the prevalence of CeD is very low (≈1%).

**Objective:**

This study aimed (1) to determine the diagnostic performance of DGP antibodies-based serologic assays in identifying CeD during the screening of the general population and (2) to compare the levels of anti-DGP antibodies among CeD patients with mild and severe degrees of enteropathy.

**Methods:**

Serology tests for DGP antibodies (DGP-IgA, DGP-IgG, and conjugate TTG/DGP antibodies) were performed on 104 serum samples of positive TTG-IgA (100 confirmed and four potential celiac patients) and a randomly selected 1,000 negative TTG-IgA serum samples collected during mass screening of children (aged 6–15 years) in 2014–2015.

**Results:**

Sera from 32 of the 1,000 TTG-IgA negative serum specimens (3.2%) tested positive for one or more of the three anti-DGP serology tests. A total of 13 of the 32 anti-DGP seropositive patients had persistent positive results on follow-up samples in 2020 (1.3%). Eight of the 13 underwent endoscopy with biopsies, and only two had confirmed CeD (both DGP-IgG positive) (0.2%). The sensitivity and specificity of the serology assays were as follows: DGP-IgA (62.7%, 40%), DGP-IgG (80.4%, 100%), and conjugate TTG/DGP (96%, 10%). Based on receiver operating characteristic curves, the area under the curve for DGP-IgG (0.919; 95% CI −0.00406 to 0.114) was comparable to TTG-IgA (0.974; 95% CI 0.924–0.995) (*P* = 0.0679). Titers of antibodies to DGPs were significantly higher in children with severe intestinal damage than in those in children with mild lesions (*P* < 0.001).

**Conclusion:**

The TTG-IgA assay remains the most reliable screening serology test for CeD in mass screening studies. The performance of TTG-IgA has improved marginally by adding DGP-IgG to the mass screening protocol. In CeD patients detected by mass screening, the anti-DGP antibody titer was significantly higher among patients with a severe degree of enteropathy as compared to the group with mild enteropathy.

## Introduction

The majority of the high sensitivity and specificity data (>90%) for celiac disease (CeD) serologic tests had been generated from studies conducted in populations with a high pretest probability of CeD (prevalence of CeD is >50% of the population under study) (1). The sensitivity of anti-tissue transglutaminase (TTG) antibody [anti-TTG immunoglobulin A (IgA)]-based tests varied between 52.9% and 82.3% when they were used to identify CeD patients in lower-risk populations (prevalence of CeD is between 5% and 10%) (2, 3), and the tests may perform even less well in the general population where the prevalence of CeD is very low (≈1%). Although anti-TTG-IgA is the most popular and well-established serology test to screen for CeD in population screening studies, the performance in this population with a low pretest probability of CeD suggests that its use alone might not be a wise strategy and that combining with another serology test could be a better strategy to accurately identify celiac patients that needs endoscopy.

Celiac serology has evolved, with the identification of newer antibodies against deamidated gliadin peptides (anti-DGP, IgA, and IgG types) with sensitivity and specificity in detecting CeD that are equivalent to IgA-TTG (4–8). The excellent performance of the CeD serology assays opens the possibility that these tests can be used, alone or in combination, not only to accurately identify celiac patients that need endoscopy but also as a substitute for intestinal biopsies in a selected group of patients. Our aims in this cross-sectional prospective study were as follows: (1) to determine the diagnostic performance of individual DGP antibody-based serologic assays to identify CeD patients in a mass screening study of the general population and (2) to compare the levels of anti-DGP antibodies among CeD patients with mild and severe degrees of enteropathy.

### Patients and methods

### Study design and population

The present study is a sub-study of a cross-sectional mass screening study of CeD among school-aged Saudi children, the details of which have been published elsewhere (9). We reported a prevalence of 1.5% (one CeD case per 66 healthy Saudi children) (9).

The present research project consisted of two phases:

A. Cross-sectional screening for CeD using DGP-IgA, DGP-IgG, and conjugate DGP/TTG (Elisa serology assays) was performed on two sets of stored serum samples collected in 2014–2015 during the mass screening study:
  (1) Group 1
    This group comprised 1,000 serum specimens randomly selected from the 7,709 TTG-IgA negative sera for apparently healthy children: 299 males and 601 females with a mean age of 11.7 ± 2.9 years.
  (2) Group 2
    This group comprised 104 serum samples of positive TTG-IgA collected within 1 month prior to endoscopy. These specimens belong to 100 celiac patients (90 were biopsy-proven and 10 were based on ESPGHAN non-biopsy criteria), with a mean age of 11.4 ± 2.6 years (80 females), and four potential celiac patients.
B. A prospective phase of the study that included a follow-up serology testing [TTG-IgA, DGP-IgA, DGP-IgG, conjugate DGP/TTG, and endomysial antibody (EMA)] performed in 2019 on sera collected from the students whose sera tested positive for DGP-IgA, DGP-IgG, and/or conjugate DGP/TTG but negative for TTG-IgA during the cross-sectional phase in 2014 (Group 3). The same serology kits were used on both the first and second samples. The students who again tested positive for DGP-IgA, DGP-IgG, and/or conjugate DGP/TTG on the repeat samples were offered endoscopy.

### Study procedures

– The five serology assays were performed, according to the manufacturer’s guidelines set by Inova Diagnostics as described before (9, 10), by the same laboratory personnel who were involved in the mass screening study. The anti-TTG/DGP-IgA and IgG ELISA test is designed to detect simultaneously IgA and IgG antibodies against a mixture of TTG and DGP in human serum. The samples were interpreted as negative (no DGP and TTG IgG or IgA antibodies) [<20 units] or positive (presence of DGP and/or TTG IgG and/or IgA antibodies) [≥20 units]. For the anti-DGP-IgA and IgG assay, the test was considered positive if ≥25 units. For TTG-IgA, values of >20 units were considered positive.
– During the procedure, six biopsies were obtained from the second and third parts of the duodenum and two biopsies from the duodenal cap. The histopathological features were graded according to the Marsh classification (11) and the modified version introduced by Oberhuber (12). For simplicity, patients with Marsh 2 and 3a were categorized into a mild intestinal damage category, and patients with Marsh 3b and 3c were categorized into a severe intestinal damage group.

Diagnosis of CeD was established if a positive serologic test was accompanied by histopathological features consistent with CeD (Marsh class ≥ 2) or based on the ESPGHAN non-biopsy criteria (5). “Potential CeD” defines patients with positive TTG-IgA and marsh classification histopathology grade 0/1 on intestinal biopsies. The details of the biopsy protocol during the mass screening study have been published elsewhere (6). In brief, the criteria to undergo intestinal biopsies were (1) children with a TGA-IgA titer > 60 U/L and (2) children with borderline positive TGA-IgA titers (20–60 U/L) and positive EMA-IgA.

### Ethical considerations

The local institutional review board (IRB no. 16-206) has approved the study proposal. The parents of the participating students have signed an informed consent.

### Statistical analysis

From the receiver operating characteristic (ROC) curves (excluding IgA-deficient patients), we identified the area under the ROC curve (AUROC). The difference in median values of the serology test titers between the mild and severe intestinal damage groups was evaluated by the Mann–Whitney *U*-test. Statistical significance was inferred at *P*-values <0.05 for all comparisons. All data were entered and analyzed through the statistical package SPSS 25 (SPSS Inc., Chicago, IL, USA).

## Results

### Screening for CeD on previously collected and stored serum specimens

**Figure 1:**
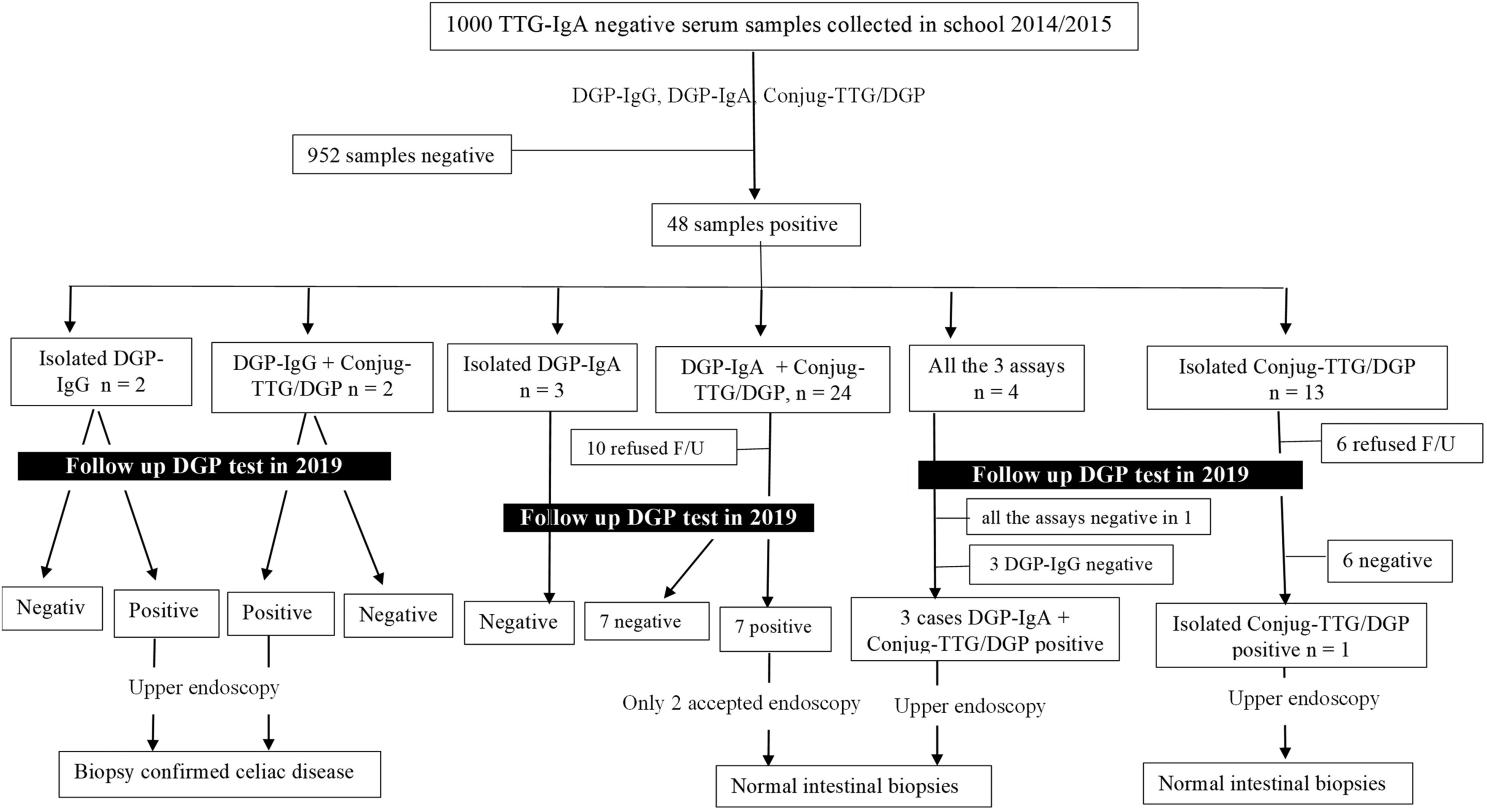
Flowchart that summarizes the results and outcomes of the 1,000 tissue transglutaminase-IgA negative patients tested for the anti-deamidated gliadin peptide antibodies. F/U, follow-up.

(1) Group 1 (1,000 serum samples negative for TTG-IgA)
  Sera from 48 (4.8%) students tested positive for one or more of the three anti-DGP serology tests (mean 11.8 ± 2.6 years, 39 females), as outlined in Figure 1. The age, gender, and levels of the three serology tests in the sera of 48 students are shown in Supplementary Table S1. None of the 48 positive sera was deficient in total IgA.
(2) Group 2 (100 CeD and four potential celiac patients)
  The results of anti-DGP testing are shown in Supplementary Figures S1A–D. The rate of missed CeD patients in the screening phase was 37% for DGP-IgA, 20% for DGP-IgG, 3% for conjugate TTG/DGP, and 13% for EMA (Supplementary Figures S1A–D). The single CeD patient with total IgA deficiency tested positive for DGP-IgG (titer = 77 U/L).
(3) Group 3 (follow-up of the 48 children with positive DGP-based serology tests but negative for TTG-IgA)
  The mean age at retesting was 15.8 ± 2.6 years. Thirty-two of the 48 anti-DGP seropositive patients accepted to undergo follow-up serology testing (Supplementary Table S1). Nineteen of the 32 anti-DGP seropositive patients turned negative on follow-up serum samples (Figure 1). Of the 13 persistently positive anti-DGP patients, five declined and eight agreed to undergo endoscopy. Only two out of the eight patients (one with isolated positivity for DGP-IgG [=56 U/L] and the other one positive for both DGP-IgG [44 U/L] and conjugate TTG/DGP [54 U/L]) had biopsy-confirmed CeD (Marsh 3A and Marsh 2 histology, respectively). The former patient had EMA positivity, and the latter was negative for EMA. Both patients carry heterozygous DQ8 molecules.

### Diagnostic performance of the five individual serology tests

By using the cut-off level set by the manufacturer for a positive result, the TTG-IgA assay detected 103 of 106 CeD children (97.1%) and missed three children: two were positive for DGP-IgG, including one with IgA deficiency. The conjugate TTG/DGP test detected 98 of 102 (96%) CeD children (Supplementary Figure S2). A summary of the diagnostic performance of the five serology tests in screening for CeD is shown in Table 1, and the details are shown in Supplementary Table S2.

**Table 1:** Performance of the serologic assays.

	Sensitivity (%)	Specificity (%)	Positive predictive value (%)	Negative predictive value (%)
TTG-IgA	97.1	60	96.2	66.6
DPG-IgG	80.4	100	100	33.3
DGP-IgA	62.7	40	91.4	9.5
Conjugate TTG/DGP	96	10	91.6	20
EMA	87.7	80	96	38
TTG-IgA + DGP-IgG	100	60	96.4	100
TTG-IgA + DGP-IgA	97.1	0	91.15	0
TTG-IgA + conjugate TTG/DGP	98.1	0	91.2	0
TTG-IgA + EMA	97.1	60	96.2	66.6

**Figure 2:**
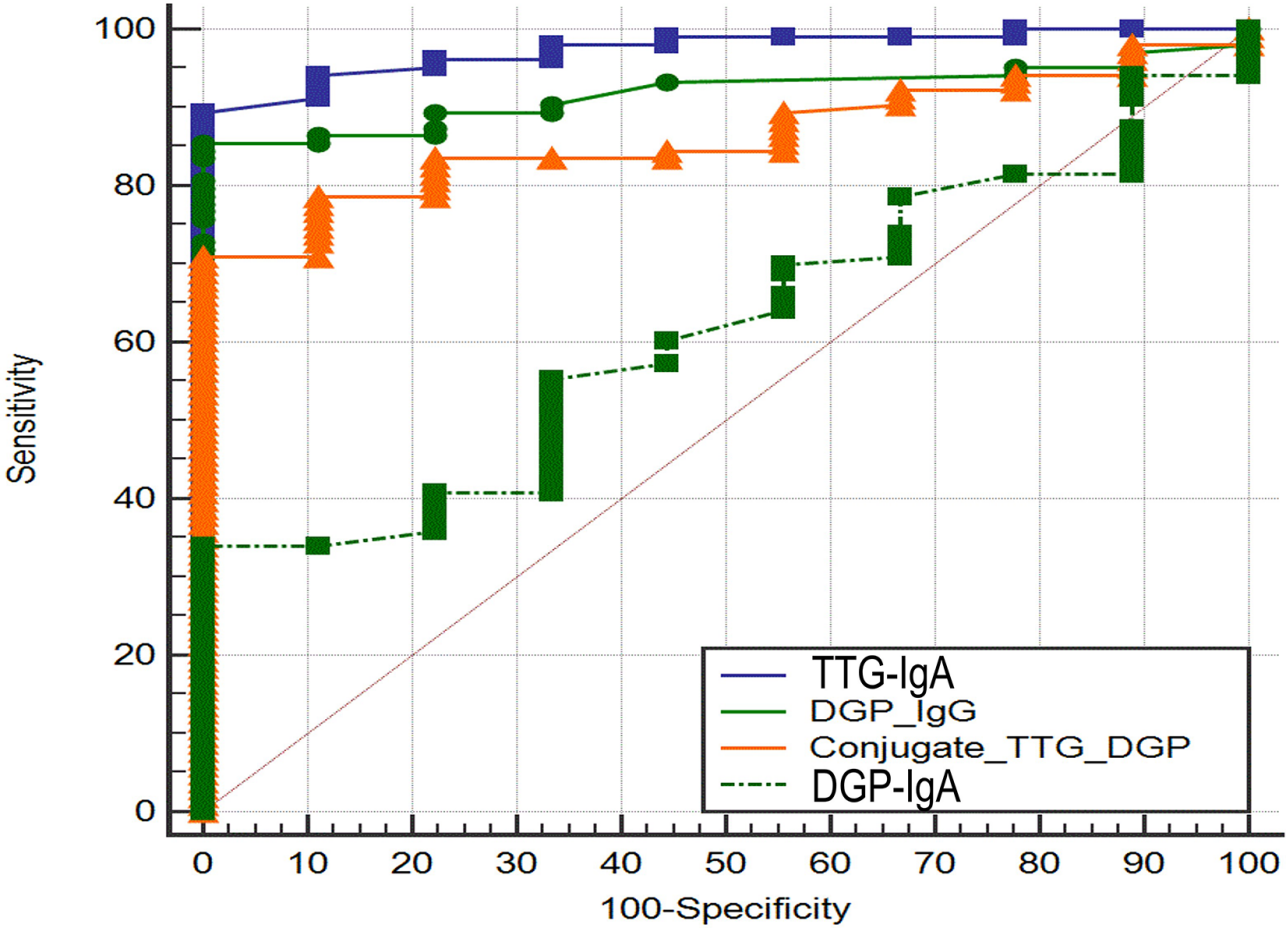
Receiver operating characteristic curve analysis of tissue transglutaminase-IgA and deamidated gliadin peptide-IgG.

The AUROC for TTG-IgA (0.974; 95% CI 0.924–0.995) and DGP-IgG (0.919; 95% CI −0.00406 to 0.114) was not significantly different (*P* = 0.0679). The area under the ROC curve for TTG-IgA was significantly higher than AUROC for DGP-IgA (0.616; 95% CI 0.519–0.706; *P* < 0.0001) and conjugate TTG/DGP (0.861; 95% CI 0.783–0.919; *P* = 0.003) (Figure 2).

### The diagnostic performance of various combinations of CeD-specific antibodies

We calculated the sensitivity, specificity, positive predictive values (PPVs), and negative predictive values (NPVs) with the condition that a given combination was considered positive if one assay was above the cut-off values and the combination was considered negative if both were concordantly below the cut-off values. The combination of TTG-IgA and DGP-IgG resulted in 100% sensitivity via the detection of three CeD patients missed by TTG-IgA: one child with IgA deficiency and two patients of CeD with isolated DGP-IgG positivity (Table 1). The addition of either DGP-IgA or conjugate TTG/DGP assays did not improve the diagnostic performance of TTG-IgA; on the contrary, the positivity of DGP-IgA or conjugate TTG/DGP when TTG-IgA is negative could have resulted in six and nine unnecessary endoscopies, respectively (i.e., negative TGA-IgA but false-positive DGP). Four CeD patients had positive TTG-IgA and negative EMA and DGP-IgG.

### Comparison of the titers of antibodies to TTG and DGPs between the mild and severe intestinal damage groups

**Figure 3:**
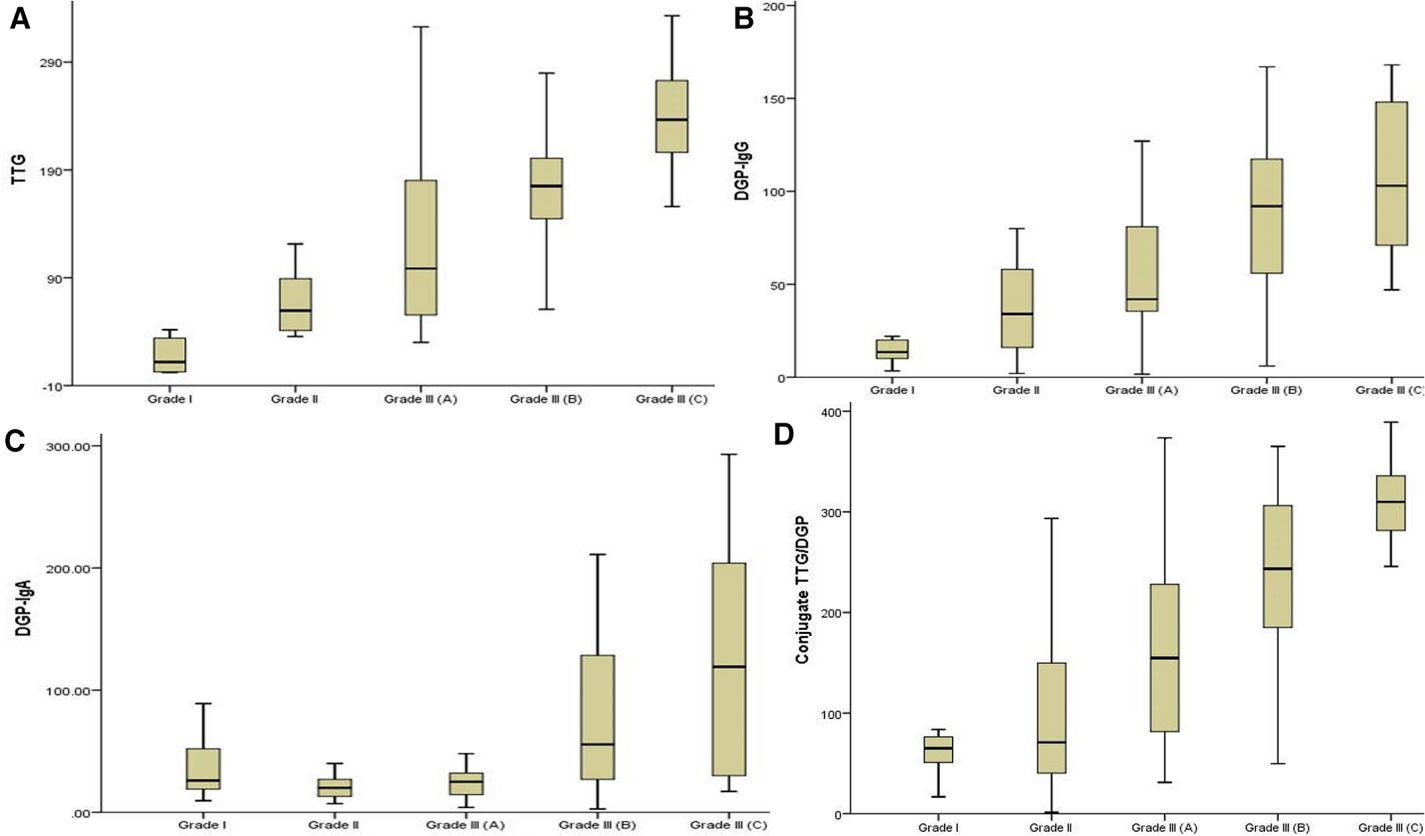
(**A–D**) A comparison of the four serology antibody titers among the mild and severe enteropathy groups.

Of the 92 biopsy-confirmed CeD children, 42 (45.6%) had severe intestinal damage [Marsh 3b (*n* = 27) and 3c (*n* = 15)] and 50 (54.4%) showed mild intestinal damage [Marsh 2 (*n* = 21) and 3a (*n* = 29)]. Titers of antibodies to DGPs and TTG were significantly higher in children with severe intestinal damage than in those with mild lesions (Figures 3A–D), with this trend more marked for TTG-IgA, conjugate TTG/DGP, and DGP-IgG, and less for DGP-IgA. For DGP-IgG, the median titer was 80 U/L ([60.50–126.00) in marsh grade 3b and 3c children and 42 U/L (23.25–72.75) in the children with mild intestinal damage (*P* < 0.001).

### Evaluation of the performance of the ESPGHAN criteria for the non-biopsy diagnosis of children with CED detected during mass screening of the general population

There were 34 children (36%) with anti-TTG-IgA titer > 10× upper limit of normal (ULN); all had histological changes (Marsh 3a–3c) consistent with a definitive diagnosis of CeD. All of the 34 children in this group were EMA-positive. Thirty of the 82 DGP-IgG positive CeD children had titer ≥ 90 U/L, and all showed villous atrophy on duodenal biopsies. Fourteen of the 30 children (47%) met the ESPGHAN criteria for the non-biopsy diagnosis of CeD (i.e 14 of the 30 children had DGP-IgG > 90 U/L and also had TTG-IgA > 10× ULN).

## Discussion

Our study highlights several observations. First, our results show that TTG-IgA is still the most robust and reliable tool for identifying CeD in a mass screening situation. Second, by adding DGP-IgG to the mass screening protocol, we found that the performance of TTG-IgA has improved marginally as DGP-IgG could rarely recognize CeD patients that TTG-IgA has missed; this finding is in agreement with several studies that showed that anti-DGP antibodies identified CeD patients negative for TTG-IgA (10–13). In line with previous studies on screening for CeD among high-risk groups, we demonstrate here that the diagnostic performance of DGP-IgG antibodies in the mass screening of the general population is higher than that of the DGP-IgA and conjugate TTG/DGP assays that did not add any advantage to the TTG-IgA assay. Also, we found that DGP-IgG levels > 90 U/ml (≈4× ULN) were 100% predictive of enteropathy consistent with CeD diagnosis.

Previous reports compared the diagnostic performance of celiac serology antibodies in selected groups of patients, mostly those with symptomatic disease or high-risk groups. Lammi et al. (13) analyzed the performance of the anti-DGP assay as compared to TTG-IgA in the diagnosis of 92 children with biopsy-confirmed CeD and found that the anti-DGP assay (IgG and IgA) is as useful as the TTG-IgA assay for detecting CeD in children with sensitivity and specificity > 90% with the area under the ROC curve of 0.99 for both classes of antibodies. However, the study group did not represent the general population as the study sample was a mix of 44 children clinically suspected of CeD and 48 children diagnosed during the screening of genetically susceptible individuals monitored prospectively for the development of type 1 diabetes-associated autoantibodies and TTG-IgA. This obvious selection bias might explain the high sensitivity and specificity of the DGP-based assay. According to our data, 38% and 20% of the 102 CeD patients would have been missed by a screening approach based on DGP-IgA and DGP-IgG as the single initial investigation, respectively. The conjugate TTG/DGP test detected 96% of the 102 CeD patients; however, it has an unacceptable low specificity (10%). Six patients with false-positive DGP-IgA and conjugate TTG/DGP led to unnecessary endoscopies. These data indicate that none of the DGP-based assays is appropriate to be the single first-line screening serology test in the general population. Our study definitively confirms the higher sensitivity of the TTG-IgA assay in comparison with DGP-based assays to detect CeD in the screening studies of the general population.

Sensitivity is not only crucial for finding new patients but also for evaluating whether TTG-IgA seropositivity would correctly select patients for endoscopy and biopsies and avoid unnecessary procedures, whereas specificity is the critical parameter. The 60% specificity of TTG-IgA in our mass screening study is relatively low and would be unacceptable for screening the general population. This potential limitation could hamper efforts to initiate population mass screening. Given the excellent specificity of the persistently positive DGP-IgG assay in our study (100%), and in addition to its ability to identify a single CeD patient with total IgA deficiency, it could be used in combination with TTG-IgA test in initial screening of general population without the need to measure total IgA. Using this combination of the two tests can increase the sensitivity without lowering the specificity. Earlier studies among high-risk populations have concluded that DGP-IgG in combination with TTG-IgA has higher sensitivity than that with TTG-IgA alone (14–16), which is also in concordance with our findings in low-risk populations. However, the adoption of this strategy in mass screening of the general population entails a higher cost than the combination of TT-IgA and total IgA. Is it more cost-effective to add a DGP-IgG assay to the initial TTG-A-IgA-based diagnostic workup vs. initial TTG-IgA + total IgA in screening the general population? Mass screening studies investigating the cost-effectiveness of these two approaches in large populations are needed to answer this question.

Transient TTG-IgA or DGP antibody seropositivity has been observed in several studies (17–19). In the present study, 19 out of 32 anti-DGP seropositive students turned negative on follow-up serum samples 5 years later. Similarly, in our mass screening study, 50 of the 221 positive TTG-IgA students on the first blood sample had transiently positive TTG-IgA on repeat samples 6–12 months later (9). It is possible that seropositivity for anti-DGP and TTG-IgA in the general pediatric population may be a transient phenomenon and does not necessarily predict the clinical onset of CeD and should not warrant moving directly to endoscopy and biopsy as many of the DGP values, especially in borderline positive titers (i.e., 1–3 times the ULN), spontaneously decreased without implementation of a gluten-free diet (20). In this line, the ESPGHAN guidelines recommend that asymptomatic children with mildly elevated TTG-IgA should be followed up to evaluate the trend in serological testing rather than undergoing an intestinal biopsy because it has been found that the PPV of isolated mildly elevated TTG-IgA is poor (5). We believe that the same holds true in the healthy pediatric general population who are found to have isolated DGP-IgG seropositivity as two of eight patients in our cohort persisted to have positive DGP-IgG serology on follow-up and proved to be diagnosed with CeD. Our finding is corroborated by several studies that observed that DGP-IgG could precede the appearance of TTG-IgA in some celiac patients (19, 21–23). On the other hand, other studies showed that isolated positive DGP-IgG has a low diagnostic yield for CeD (24, 25); hence, we think that monitoring isolated positive DGP-IgG patients should be a reasonable approach. In contrast to DGP-IgG, our data showed that isolated persistently positive DGP-IgA or conjugate TTG/DGP tests have a very poor PPV for CeD in the general pediatric population.

The accuracy of the celiac serology assays with their PPVs and the limitations associated with histological diagnosis of CeD in clinical practice (26, 27) have attracted researchers to investigate their use as an alternative to biopsy to overcome these challenges. In a previous study, we showed that TTG-IgA titer correlated with enteropathy in CeD patients detected by mass screening, and we proposed that the no-biopsy approach to diagnose CeD may be of use in screening-detected children from the general population with a TTG-IgA titer > 10× ULN given EMA positivity (28). Also in our present study, we observed a statistically significant difference in anti-DGP titers between the mild and severe intestinal damage groups (median titer of 42 U/L vs. 80 U/L, respectively; *P* < 0.001). Thirty of the 82 DGP-IgG positive CeD children had titer > 90 U/L, and all showed villous atrophy on duodenal biopsies. In line with the relevance of CeD antibody titers stated by ESPGHAN for TTG-IgA (5) and our finding that high DGP-IG titers are closely related to severe intestinal damage, a high DGP-IgG titer > 90 (≈4× ULN) may represent a diagnostic alternative to biopsy. This observation needs to be confirmed in other large prospective mass screening studies.

Our study suffered from ascertainment bias as the vast majority of patients were selected for biopsy because they had positive TTG-IgA serology and the resultant sensitivity could be falsely high. In addition, our results were obtained utilizing only one TTG-IgA assay and thus cannot be directly generalized due to a lack of standardization between the commercial tests. Another limitation was the relatively small sample of 1,000 children from the general population screened by DGP-IgG and the resultant small number of the isolated positive DGP-IgG children who underwent upper endoscopy and intestinal biopsies; therefore, the results of the performance of the DGP-IgG assay obtained in our study should be interpreted with caution. On the other hand, there are several strengths of our study. First, we used a prospective study design, with a similar protocol for the diagnostic process for all the screened TTG-IgA-positive children. Second, all the 1,000 screened TTG-IgA negative specimens in the mass screening study were analyzed 4–5 years later by the same laboratory and same test kit. Because all the 1,000 participants were unaware of the DGP determination on the first specimen, we were able to study the development of anti-DGP antibody levels over 4–5 years. Another strength of our study is that TTG-IgA measurements were performed in combination with the assessment of EMA, DGP assays, and biopsy specimens at the same time as the second serology assessment. Furthermore, multiple biopsies were obtained from both the proximal and the distal duodenum, and a single expert GI pathologist, blind to the serology titer results, evaluated all biopsies thus minimizing the interobserver variability.

In conclusion, the TTG-IgA assay remains the most reliable screening serology test for CeD even in a very low pretest probability situation. The performance of DGP-IgG in children not preselected by TTG-IgA testing must be resolved in prospective studies. If the high specificity of DGP-IgG observed in our study is confirmed in large prospective mass screening studies, the combination of TTG-IgA and DGP-IgG protocol could be recommended to screen for CeD among the general pediatric population.

## Data Availability

The original contributions presented in the study are included in the article/**Supplementary Material**, further inquiries can be directed to the corresponding author.

## Supplementary material

Supplementary Figure S1 (**A–D**) The results of anti- deamidated gliadin peptides and endomysial antibody testing on the 104 positive tissue transglutaminase-IgA sera.

Supplementary Figure S2 Antibody levels of the 112 children measured by the four serology tests.
