# Supplementary material for: Performance of deamidated gliadin peptide antibodies as first screening for celiac disease in the general pediatric population
Source: Front Pediatr. 2023 Nov 21;11:1279825. doi: 10.3389/fped.2023.1279825 (PMC10703185; doi:10.3389/fped.2023.1279825)
Supplement: Supplementary file 1 [file Table1.pdf]

Supplementary table 1: Characteristics of the 48 cases with negative TTG-IgA and positivity of anti-DGP serology tests

Case	Age (year)	Sex	First serum sample at school (2014)			Second serum sample 5 years later					biopsies
			DGP-IgG	DGP-IgA	TTG/DGP	DGP-IgG	DGP-IgA	TTG/DGP	EMA	TTG-IgA	
**1**	14	F	6.5	40.9	63.2	1	2.7	12	-ve	3.7	-
**2**	7	F	3.1	7	49.4	1.5	18	21	-ve	7	-
**3**	12	F	3.9	4.6	28.6	3.6	5.1	19	-ve	4.7	-
**4**	7	F	10.5	30.3	34.7	4.5	11.6	21	-ve	5.8	-
**5**	14	F	3.3	30.7	40.7	1	6.4	17	-ve	4	-
**6**	15	F	2	19.6	28.7	-	-	-	-		
**7**	16	F	2.75	51.8	44.1		34	48	-ve	3	Normal
**8**	13	F	2.01	301.9	245.7	1.3	200	220	-ve	6	Normal
**9**	13	F	6.6	28.6	35.8	1.2	3.2	9	-ve	3.6	-
**10**	14	F	5.9	16.6	27.9	-	-	-	-		
**11**	16	F	16.4	14.2	26.4	23.1	9.2	17	-ve	5.7	-
**12**	13	F	1.4	56.7	35	-	-	-	-		
**13**	13	F	3.4	16.6	27.2	2.5	10	18	-ve	11	-
**14**	13	F	1.7	52.2	23.4	4	18	22	-ve		-
**15**	13	F	2.52	30.8	39	0.7	16.5	21	-ve	10.3	-
**16**	10	F	5	37.5	52.3	-	-	-	-		
**17**	8	F	4.9	36.117	65.86	3.1	13.5	23	-ve	21.2	-
**18**	10	F	2.5	19.75	31.6	0.9	2.7	7	-ve	2.2	-
**19**	10	F	20.9	8.78	28.7	-	-	-	-		
**20**	12	F	19	21.3	28.3	-	-	-	-		
**21**	15	F	21.4	4.75	27.5	-	-	-	-		
**22**	9	F	21.4	18.4	38.2	-	-	-	-		
**23**	9	F	27.5	45	29	20.4	6.7	18	-ve	2.8	-
**24**	8	F	2.1	60.4	35.6	4	46	53	-ve	3	Not done
25	10	F	50.2	3.3	10	18	5	20	-ve	1.8	-
**26**	15	F	29	42.2	49	14	39	44	-ve	2	Normal
**27**	15	F	81.2	37.8	86.6	21	27	45	-ve	1.9	Normal
**28**	14	F	16.2	52	34.3	-	-	-	-		
**29**	10	F	4.4	31.7	14.5	2.3	14.4	19	-ve	3.3	-
**30**	13	F	48.9	21	57	44	24	54	-ve	2	Celiac
**31**	16	F	31.6	15.4	45.3	16	9	23	-ve	17.5	-
**32**	15	F	3.1	29.3	26.9	-	-	-	-		
**33**	14	F	34	12	19	56	20.89	22	+ve	19.2	Celiac
**34**	9	F	2.9	46.3	14.2	5	16	22	-ve	4	-
**35**	14	F	5.4	46.7	38.2	-	-	-	-		
**36**	9	F	15.5	14.3	38.5	16.1	6.6	23	-ve	3.3	-
**37**	13	F	1.5	42.2	26	3.5	6	22	-ve	3	-
**38**	10	F	23.1	76.6	75.3	7.6	35.4	68	-ve	4.9	Not done
**39**	9	F	54.5	59.7	81	13	44	65	-ve	4	Normal
**40**	8	M	1.6	110.1	69.2	2	59	31	-ve	4.5	Not done
**41**	9	M	9.27	44	45.5	-	-	-	-		
**42**	7	M	3.9	64.9	74.1	-	-	-	-		
**43**	15	M	2.82	62.6	65	1.6	45	56	-ve	13.5	Not done
**44**	14	M	8.16	60.5	58.7	-	-	-	-		

**45**	12	M	19.4	31.6	45.1	-	-	-	-		
**46**	9	M	16.6	84.1	38.1	5.8	79	58	-ve	6	Not done
**47**	12	M	2.3	45.6	31.9	-	-	-	-		
**48**	12	M	18.7	19.1	99.6	6.4	18	85	-ve	3.7	Normal
