# Supplementary material for: Performance of deamidated gliadin peptide antibodies as first screening for celiac disease in the general pediatric population
Source: Front Pediatr. 2023 Nov 21;11:1279825. doi: 10.3389/fped.2023.1279825 (PMC10703185; doi:10.3389/fped.2023.1279825)
Supplement: Supplementary file 2 [file Datasheet1.pdf]

1) <u>Diagnostic performance of anti-deamidated gliadin peptides IgA (DGP-IgA)</u>

2X2 table below shows the diagnostic performance of deamidated gliadin peptides -IgA serology test.

Celiac Disease (CeD)

DGP-IgA	Present	n	Absent	n	Total
Positive	True Positive	a= 64	False Positive	c= 6	a + c = 70
Negative	False Negative	b= 38	True Negative	d= 4	b + d = 42
**Total**		a + b = **102**		c + d = **10**	**112**

a: Number of cases with positive test result and CeD: true-positives; b: Number of cases with positive test result and without CeD: false-positives; c: Number of cases with negative test result and CeD: false-negatives; d: Number of cases with negative result and without CeD: true-negatives.

Statistic	Formula	Value	95% CI
Sensitivity	$\frac{a}{a+b}$	62.7%	52.6% to 72.1%
Specificity	$\frac{d}{c+d}$	40%	12.1% to 73.7%
Positive Predictive Value	$\frac{a}{a+c}$	91.4%	86.3% to 94.7%
Negative Predictive Value	$\frac{d}{b+d}$	9.5 %	4.52% to 18.98%

2) <u>Diagnostic performance of anti-deamidated gliadin peptides IgG (DGP-IgG)</u>
2X2 table below shows the diagnostic performance of DGP-IgG serology test.

Celiac Disease

DGP-IgG	Present	n	Absent	n	Total
Positive	True Positive	a= 82	False Positive	c= 0	a + c = 82
Negative	False Negative	b= 20	True Negative	d= 10	b + d = 30
**Total**		a + b = **102**		c + d = **10**	**112**

Statistic	Formula	Value	95% CI
Sensitivity	$\frac{a}{a+b}$	80.4%	71.3% to 87.5%
Specificity	$\frac{d}{c+d}$	100%	69.15% to 100%
Positive Predictive Value	$\frac{a}{a+c}$	100%	
Negative Predictive Value	$\frac{d}{b+d}$	33.3 %	25.2% to 42.5%

3) <u>Diagnostic performance of conjugate anti-tissue transglutaminase/ deamidated gliadin peptides (conjugate TTG/DGP)</u>

2X2 table below shows the diagnostic performance of conjugate TTG/DGP serology test.

	Celiac Disease				
**Conjugate TTG/DGP**	**Present**	**n**	**Absent**	**n**	**Total**
**Positive**	True Positive	a= 98	False Positive	c= 9	a + c = 107
**Negative**	False Negative	b= 4	True Negative	d= 1	b + d = 5
**Total**		a + b = **102**		c + d = **10**	**112**

Statistic	Formula	Value	95% CI
Sensitivity	$\frac{a}{a+b}$	96%	90.2% to 98.9%
Specificity	$\frac{d}{c+d}$	10%	0.25% to 44.5%
Positive Predictive Value	$\frac{a}{a+c}$	91.6%	89.82% to 93%
Negative Predictive Value	$\frac{d}{b+d}$	20 %	2.3% to 66.9%

4) Diagnostic performance of tissue transglutaminase-IgA (TTG-IgA)

2X2 table below shows the diagnostic performance of TTG-IgA serology test.

Celiac Disease

TTG-IgA	Present	n	Absent	n	Total
Positive	True Positive	a= 103	False Positive	c= 4	a + c = 107
Negative	False Negative	b= 3	True Negative	d= 6	b + d = 9
**Total**		a + b = **106**		c + d = **10**	**116**

Statistic	Formula	Value	95% CI
Sensitivity	$\frac{a}{a+b}$	97.1%	91.9% to 99.4%
Specificity	$\frac{d}{c+d}$	60%	26.2% to 87.8%
Positive Predictive Value	$\frac{a}{a+c}$	96.2%	92.3% to 98.2%
Negative Predictive Value	$\frac{d}{b+d}$	66.6%	37% to 87.2%

5) Diagnostic performance of endomyseal antibody (EMA)

2X2 table below shows the diagnostic performance of EMA serology test.

Celiac Disease

EMA	Present	n	Absent	n	Total
Positive	True Positive	a= 93	False Positive	c= 2	a + c = 95
Negative	False Negative	b= 13	True Negative	d= 8	b + d = 21
**Total**		a + b = **106**		c + d = **10**	**116**

Statistic	Formula	Value	95% CI
Sensitivity	$\frac{a}{a+b}$	87.7%	79.9% to 93.3%
Specificity	$\frac{d}{c+d}$	80%	44.4% to 97.5%
Positive Predictive Value	$\frac{a}{a+c}$	96%	93.% to 99.38%
Negative Predictive Value	$\frac{d}{b+d}$	38 %	25.3% to 52.7%
