# Supplementary figures and images for: Performance of deamidated gliadin peptide antibodies as first screening for celiac disease in the general pediatric population
Source: Front Pediatr. 2023 Nov 21;11:1279825. doi: 10.3389/fped.2023.1279825 (PMC10703185; doi:10.3389/fped.2023.1279825)

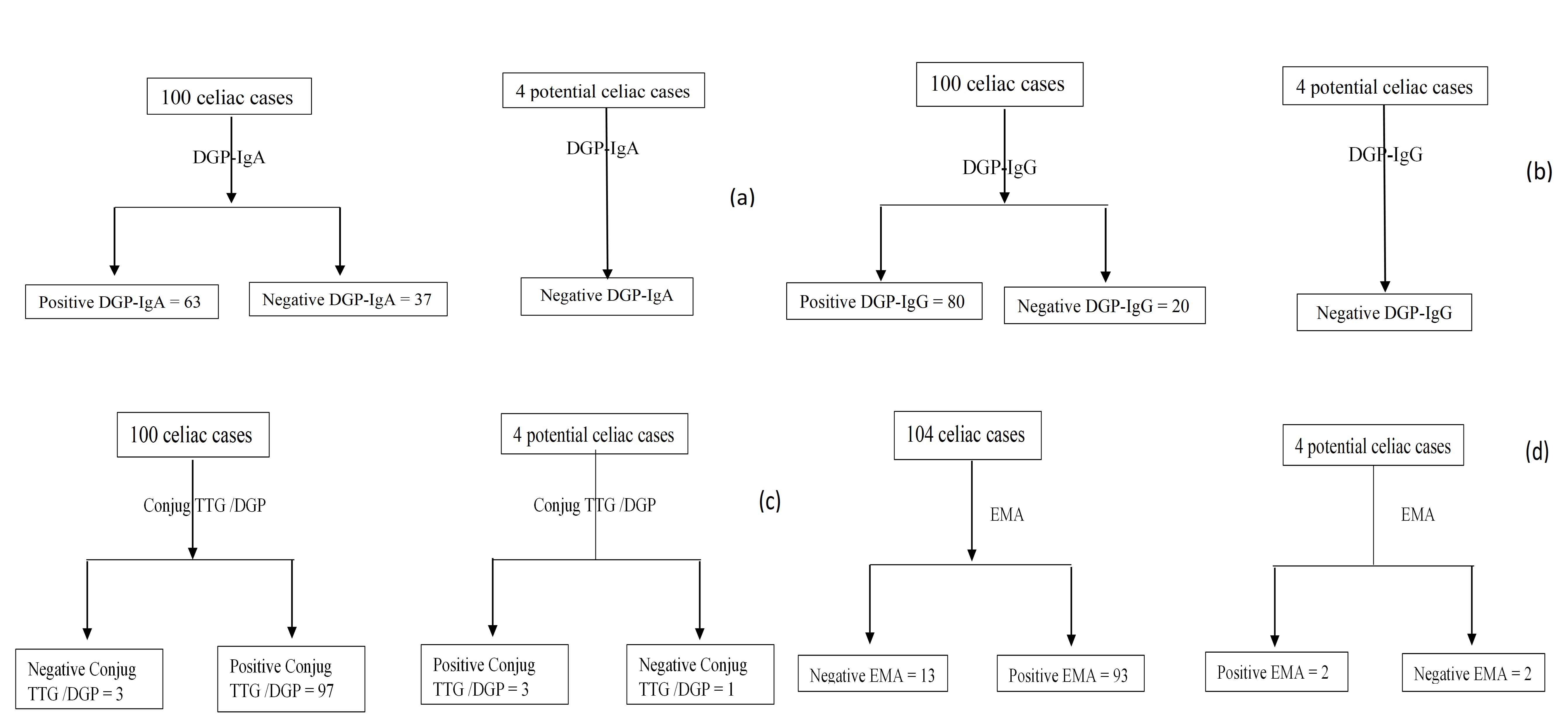

Supplement: Supplementary Figure S1 — (A–D) The results of anti- deamidated gliadin peptides and endomysial antibody testing on the 104 positive tissue transglutaminase-IgA sera. [file Image1.jpeg]

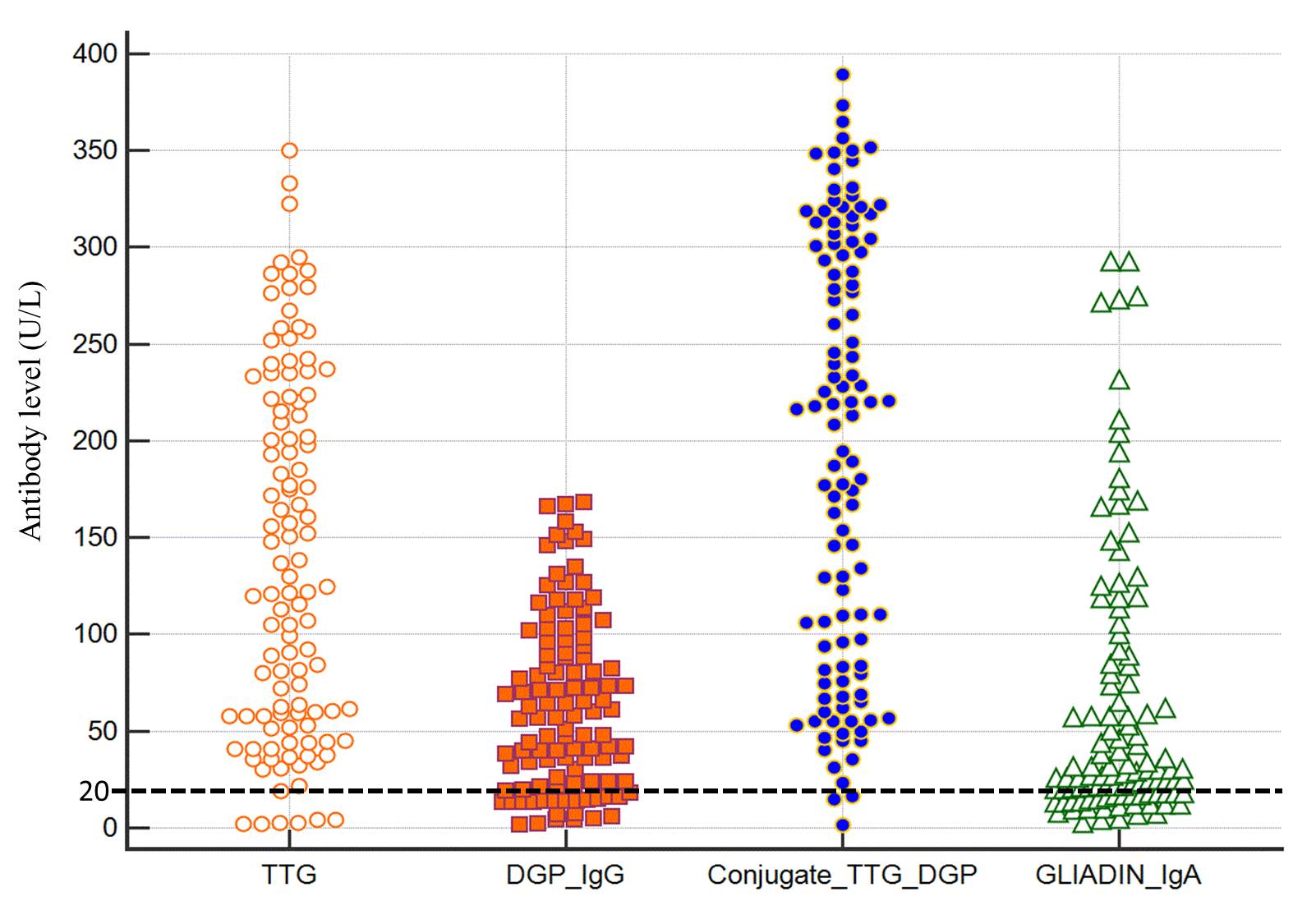

Supplement: Supplementary Figure S2 — Antibody levels of the 112 children measured by the four serology tests. [file Image2.jpeg]
